# Ideal Outcome After Pancreatoduodenectomy: A Transatlantic Evaluation of a Harmonized Composite Outcome Measure

**DOI:** 10.1097/SLA.0000000000006037

**Published:** 2023-07-21

**Authors:** Simone Augustinus, Tara M. Mackay, Bodil Andersson, Joal D. Beane, Olivier R. Busch, Elizabeth M. Gleeson, Bas G. Koerkamp, Tobias Keck, Hjalmar C. van Santvoort, Bobby Tingstedt, Ulrich F. Wellner, Caroline Williamsson, Marc G. Besselink, Henry A. Pitt

**Affiliations:** * Department of Surgery, Amsterdam UMC, Location University of Amsterdam, Amsterdam, The Netherlands; † Cancer Center Amsterdam, Amsterdam, The Netherlands; ‡ Department of Surgery, Clinical Sciences Lund, Lund University, Skåne University Hospital, Lund, Sweden; § Department of Surgery, The Ohio State University, Columbus, OH; ∥ Department of Surgery, University of North Carolina, Chapel Hill, NC; ¶ Department of Surgery, Erasmus Medical Center, Erasmus University Rotterdam, Rotterdam, The Netherlands; # DGAV StuDoQ|Pancreas and Clinic of Surgery, UKSH Campus, Lübeck, Germany; ** Department of Surgery, Regional Academic Cancer Center Utrecht, University Medical Center Utrecht and St. Antonius Hospital, Nieuwegein, The Netherlands; †† Rutgers Cancer Institute of New Jersey, New Brunswick, NJ

**Keywords:** composite outcome, ideal outcome, pancreatoduodenectomy, transatlantic

## Abstract

**Objective:**

The aim of this study is to define and assess Ideal Outcome in the national or multicenter registries of North America, Germany, the Netherlands, and Sweden.

**Background:**

Assessing outcomes after pancreatoduodenectomy among centers and countries requires a broad evaluation that cannot be captured by a single parameter. Previously, 2 composite outcome measures (textbook outcome and optimal pancreatic surgery) for pancreatoduodenectomy have been described from Europe and the United States. These composites were harmonized into ideal outcome (IO).

**Methods:**

This analysis is a transatlantic retrospective study (2018–2020) of patients after pancreatoduodenectomy within the registries from North America, Germany, The Netherlands, and Sweden. After 3 consensus meetings, IO for pancreatoduodenectomy was defined as the absence of all 6 parameters: (1) in-hospital mortality, (2) severe complications—Clavien-Dindo ≥3, (3) postoperative pancreatic fistula—International Study Group of Pancreatic Surgery (ISGPS) grade B/C, (4) reoperation, (5) hospital stay >75th percentile, and (6) readmission. Outcomes were evaluated using relative largest difference (RLD) and absolute largest difference (ALD), and multivariate regression models.

**Results:**

Overall, 21,036 patients after pancreatoduodenectomy were included, of whom 11,194 (54%) reached IO. The rate of IO varied between 55% in North America, 53% in Germany, 52% in The Netherlands, and 54% in Sweden (RLD: 1.1, ALD: 3%, *P*<0.001). Individual components varied with an ALD of 2% length of stay, 4% for in-hospital mortality, 12% severe complications, 10% postoperative pancreatic fistula, 11% reoperation, and 9% readmission. Age, sex, absence of chronic obstructive pulmonary disease, body mass index, performance status, American Society of Anesthesiologists (ASA) score, biliary drainage, absence of vascular resection, and histologic diagnosis were associated with IO. In the subgroup of patients with pancreatic adenocarcinoma, country, and neoadjuvant chemotherapy also was associated with improved IO.

**Conclusions:**

The newly developed composite outcome measure “Ideal Outcome” can be used for auditing and comparing outcomes after pancreatoduodenectomy. The observed differences can be used to guide collaborative initiatives to further improve the outcomes of pancreatic surgery.

## BACKGROUND

Clinical auditing is increasingly used to assess and improve the quality of surgical care.^1^ To accomplish this task, nationwide and multicenter registries have been established in Europe and the United States.^2–5^ In these registries, indicators such as mortality and complications are used to measure the quality of care. Monitoring and comparing the quality of specific procedures, such as pancreatic resections, requires broad assessment which may be difficult to obtain by single outcome parameters.^6,7^ Therefore, over the past years, several composite outcome measurements have been developed to evaluate outcomes of pancreatic surgery, such as textbook outcome, optimal pancreatic surgery, and benchmarks.^8–10^ While composite measures provide a more complex assessment and have known limitations, they are considered useful in providing a global picture of quality and may be better suited to measure performance.^11,12^

Textbook outcome and optimal pancreatic surgery both measure desired outcome after pancreatic resection and combine 6 variables. However, they use slightly different variables. The textbook outcome was defined as the absence of all 6 individual parameters: in-hospital mortality, severe complications (Clavien-Dindo≥3), postoperative pancreatic fistula (POPF), bile leak, postpancreatectomy hemorrhage, and readmission.^8^ Optimal pancreatic surgery was defined as the absence of in-hospital mortality, severe complications, percutaneous drainage, reoperations, prolonged length of stay (LOS) (>75th percentile), and readmission.^9^

The Global Audits on Pancreatic Surgery Group (GAPASURG) consortium aims to harmonize outcome registration for pancreatic surgery allowing for international comparison as a means to improve patient outcomes.^13^ As participants in this consortium were previously involved in developing the textbook outcome (from Europe) and optimal pancreatic surgery (from the United States), a project was initiated to harmonize both in the “Ideal Outcome” (IO) outcome measure. In this study, IO was defined and reported on in the national or multicenter registries of North America, Germany, The Netherlands, and Sweden.

## METHODS

### Study Design

This analysis was a transatlantic retrospective study of 4 registries on pancreatic surgery from the United States [American College of Surgeons National Surgical Quality Improvement Program (NSQIP): 160 centers in 2019, including several Canadian hospitals]^2^; Germany [Deutsche Gesellschaft für Allgemein- und Viszeralchirurgie- Studien-, Dokumentations- und Qualitätszentrum (DGAV StudoQ|Pancreas): 58 centers in 2019]^5^; The Netherlands [Dutch Pancreatic Cancer Audit (DPCA): 17 centers in 2019]^3,14^; and Sweden (Swedish National Pancreatic and Periampullary Cancer Registry: 6 centers in 2019).^4^ Among these, North American and German audit are multicenter (voluntary for each center) and the Dutch and Swedish registry are nationwide (mandatory for all centers). Differences among auditing and design within registries are depicted in Supplemental Digital Content 1, Table 1 (http://links.lww.com/SLA/E774). The study group on the 4 registries combined is GAPASURG (Global Audits on Pancreatic Surgery).^13^ In this analysis, all patients after pancreatoduodenectomy (PD) between 2018 and 2020 were included. The study was reported in accordance with the STROBE guidelines.^15^

### Definitions and Data Collection

After 3 online consensus meetings within the GAPASURG study group, the name “Ideal Outcome” (IO) was selected, and the definition of IO was formed. Using variables from the original optimal pancreatic surgery and textbook outcome classification (Table 1, Supplemental Digital Content 1, Table 2, http://links.lww.com/SLA/E774).^8,9^ IO included 6 variables from the original optimal pancreatic surgery and textbook outcome, that were available in the core parameter set for registries on pancreatic surgery provided by GAPASURG.^13^ IO was defined by the absence of all 6 individual parameters: in-hospital mortality, severe complications – Clavien-Dindo ≥3, POPF (grade B/C), reoperation while maintaining an acceptable postoperative LOS (≤75th percentile) with no readmission, see Table 1. Baseline variables and outcome characteristics to calculate IO were derived from the 4 registries. Baseline variables included: country, age, sex, body mass index (BMI), tumor stage, American Society of Anesthesiologists (ASA) classification, performance status [World Health Organization (WHO) or Eastern Cooperative Oncology Group (ECOG)], preoperative biliary drainage, relevant comorbidities [diabetes, heart failure, chronic obstructive pulmonary disease (COPD)], neoadjuvant chemotherapy, operative approach, type of PD (pylorus-preserving PD, pylorus resecting PD, or classic Whipple), vascular resection, operation year, and histologic diagnosis. Outcome characteristics to calculate IO were: in-hospital mortality, severe complications (Clavien-Dindo ≥3), POPF (grade B/C), reoperation, postoperative LOS, and readmission.

**TABLE 1:** Definition of IO

IO after pancreatic surgery is defined by the absence of these parameters
In-hospital mortality
Severe complications – Clavien-Dindo ≥3
Postoperative pancreatic fistula – ISGPS Grade B/C
Reoperation
Length of stay >75th percentile^*^
Readmission

* Length of hospital stay >75th percentile for the study cohort. This can be assessed in a single center cohort but also in a multicenter or nationwide cohort. For the present study, the 75th percentile was determined per audit, so for each of the 4 audits.

Differences in parameters due to the various metric systems were resolved by converting the data, ounces were converted to kilograms and inches into meters. Several variables were recategorized so that data could be combined. For example, ECOG performance status were recategorized to match the functional health status of independent (ECOG 0 or 1) or partially dependent (ECOG 2 or 3), and totally dependent (ECOG 4). In addition, tumor stage was categorized using the T-stage and N-stage according to the AJCC TNM eighth edition.^16^ Within North America and The Netherlands, the eighth edition was used to determine the T-stage and N-stage, but Germany used the Union for International Cancer Control (UICC) eighth edition,^17^ and Sweden the AJCC seventh edition.^18^ All individual components of IO were assessed during a 30-day follow-up. All in-hospital events when hospital stay exceeded >30 days were registered additionally, except within the North American registry in which only 30-day follow-up was registered. In-hospital mortality included all patients who died during the first admission; also patients who died within the hospital but after 30 days were identified as “in-hospital mortality” within the German, Dutch, and Swedish registry. Severe complications were defined according to the Clavien-Dindo classification (≥3). POPF grade B/C was defined according to the International Study Group of Pancreatic Surgery (ISGPS) in all registries.^19^ Reoperation was defined as any unplanned operating room procedure within the 30-day follow-up. To determine postoperative LOS, the median LOS per country were defined within the study period (2018–2020), and prolonged was defined as >75th percentile. Readmission was defined as any readmission (to the same or another hospital), for any reason within 30 days after the principal operative procedure.

### Statistical Analysis

Descriptive statistics were used to assess baseline characteristics. Results were reported as proportions for categorical variables, and as mean with SD or median with interquartile range for continuous variables. Normally distributed data were compared using a Student *t* test, categorical data using the χ^2^ test, and non-normally distributed data using the Mann-Whitney *U* test. Also, absolute largest difference (ALD) and relative largest difference (RLD) and between the smallest and largest outcomes of the registries were presented. Missing data on baseline characteristics were imputed by multiple imputation techniques using 10 dummy sets. Multivariable logistic regression models were performed to identify predictors of IO, variables included in this analysis were potential predictors described within the literature and adequately registered in the dataset: age, sex, ASA classification, country, BMI, performance status, relevant comorbidities (diabetes, heart failure, COPD), biliary stent placement, neoadjuvant therapy, operation year, operative approach, vascular resection, and histopathological diagnosis. All variables with a *P* value <0.2 in univariable analysis were added in the multivariable regression model. Variables were excluded through backward selection until only statistically significant variables were selected in the final multivariable model. A sensitivity analysis of complete cases was performed. All *P* values were based on a 2-sided test. A *P* value of <0.05 was considered to be statistically significant. All calculations were performed with RStudio (version 4.0.3).

## RESULTS

Overall, 21,036 patients after PD were included, of whom 13,883 (66%) were in North America, 3964 (19%) in Germany, 2188 (10%) in The Netherlands, and 1001 (5%) in Sweden. Of all included patients, 46% were female, and the median age was 68 years (interquartile range: 60–74 years). Cancer was the indication for surgery in 73% of patients, of which 59% pancreatic adenocarcinoma (PDAC), and minimally invasive PD was performed in 7% of patients (Supplemental Digital Content, Table S2, http://links.lww.com/SLA/E817 and Supplemental Digital Content, Table S3, http://links.lww.com/SLA/E818).

### Ideal Outcome

**FIGURE 1:**
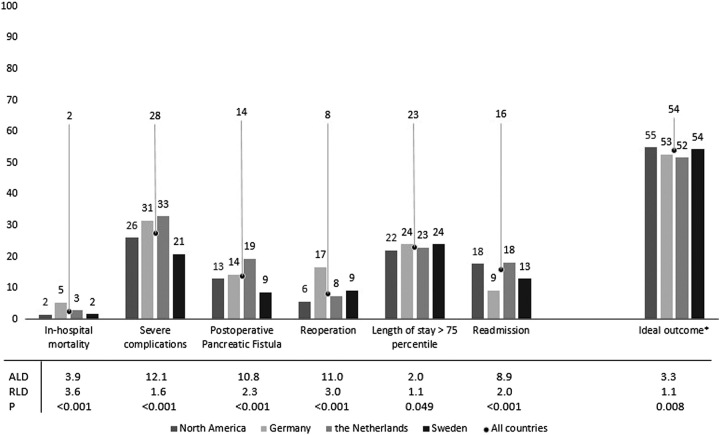
IO per country in 21,036 patients after PD. *If one of the complications occurs (in-hospital mortality, severe complications, reoperation, LOS, readmission) IO is not achieved.

IO was achieved in 54% of all patients (absence of all 6 individual components), respectively 55% in North America, 54% in Sweden, 53% in Germany, and 52% in The Netherlands (ALD: 3.3%, *P*<0.001, Fig. 1). The individual item that contributed the most in not achieving IO is severe complications (28%), followed by prolonged LOS (23%), readmission (16%), POPF (14%), reoperation (8%), and in-hospital mortality (2%). The most variation within the individual components was seen in severe complications (ALD: 12%), reoperation (ALD: 11%), POPF grade B/C (ALD: 11%), readmission (ALD: 9%), in-hospital mortality (ALD: 4%), prolonged LOS (ALD: 2%).

### Predictors of IO

Younger age, female sex, lower BMI, better performance status, preoperative biliary drainage by endoscopic retrograde cholangiopancreatography, and diagnosis of PDAC were independently associated with higher rates of IO (Table 2). On the contrary, COPD as a comorbidity, ASA score ≥3, and vascular resections were associated with worse rates of IO. No association was observed between minimally invasive surgery and IO. In the subgroup of patients with PDAC, neoadjuvant therapy was associated with higher rates of IO (odds ratio: 1.55, 95% CI: 1.42–1.69, *P*<0.001, Table 3), and country also predicted IO. Sensitivity analysis using the nonimputed set showed similar results compared with the primary analysis, with the exception of country (Sweden) (Supplemental Digital Content 1, Table 3, http://links.lww.com/SLA/E774).

**TABLE 2:** Predictors of IO

	Univariable analysis [OR (95% CI)]	*P* ^†^	Multivariable analysis^*^ [OR (95% CI)]	*P* ^‡^
Age	0.99 (0.99–0.99)	**<0.001**	0.99 (0.99–0.99)	**<0.001**
Female	1.29 (1.22–1.37)	**<0.001**	1.29 (1.23–1.37)	**<0.001**
ASA ≥ 3	0.89 (0.84–0.95)	**<0.001**	0.89 (0.83–0.94)	**<0.001**
Heart failure	0.90 (0.78–1.04)	**0.015**		
COPD	0.72 (0.64–0.82)	**<0.001**	0.76 (0.67–0.86)	**<0.001**
Diabetes mellitus	1.04 (0.97–1.10)	0.253		
Registry				
North America	Reference			
Germany	0.92 (0.85–0.98)	**0.015**		
The Netherlands	0.72 (0.80–0.96)	**0.005**		
Sweden	0.76 (0.85–1.12)	0.744		
BMI	0.99 (0.99–0.99)	**<0.001**	0.99 (0.99–0.99)	**<0.001**
Performance status				
Independent	Reference		Reference	
Partially dependent	0.61 (0.51–0.74)	**<0.001**	0.66 (0.55–0.81)	**<0.001**
Fully dependent	0.15 (0.06–0.39)	**<0.001**	0.16 (0.06–0.43)	**<0.001**
Biliary drainage				
No	Reference		Reference	
Yes – ERCP	1.34 (1.27–1.42)	**<0.001**	1.27 (1.19–1.35)	**<0.001**
Yes – PTCD	1.07 (0.83–1.22)	0.954	0.96 (0.79–1.17)	0.681
Operation year	0.99 (0.96–1.02)	0.611		
Minimally invasive surgery				
No	Reference			
Yes	1.01 (0.90–1.13)	0.880		
Other	0.82 (0.64–1.05)	**0.113**		
Vascular resection				
No	Reference		Reference	
Vein	1.07 (0.99–1.16)	**0.083**	0.91 (0.84–0.99)	**0.031**
Artery	0.87 (0.70–1.09)	0.223	0.85 (0.68–1.06)	0.130
Vein and artery	0.83 (0.68–1.01)	**0.061**	0.75 (0.62–0.92)	**0.005**
Histological diagnosis				
Pancreatic adenocarcinoma	Reference		Reference	
Distal cholangiocarcinoma	0.57 (0.50–0.64)	**<0.001**	0.56 (0.49–0.64)	**<0.001**
Ampullary carcinoma	0.72 (0.65–0.79)	**<0.001**	0.70 (0.64–0.78)	**<0.001**
Duodenal carcinoma	0.44 (0.38–0.52)	**<0.001**	0.48 (0.41–0.56)	**<0.001**
Neuroendocrine tumor	0.59 (0.52–0.68)	**<0.001**	0.59 (0.52–0.68)	**<0.001**
IPMN	0.73 (0.65–0.82)	**<0.001**	0.79 (0.71–0.90)	**<0.001**
MCN/serous cystadenoma	0.63 (0.50–0.80)	**<0.001**	0.63 (0.49–0.81)	**<0.001**
Chronic pancreatitis	0.91 (0.79–1.04)	**0.174**	0.87 (0.75–1.01)	0.067
SPN	0.58 (0.36–0.91)	**0.017**	0.46 (0.29–0.74)	**0.001**
Intestinal adenoma	0.42 (0.27–0.65)	**<0.001**	0.42 (0.27–0.65)	**<0.001**
Other	0.57 (0.52–0.63)	**<0.001**	0.57 (0.52–0.64)	**<0.001**

* Multivariable analysis in 21,036 patients, no deletions due to missing values (on the imputed set).

† Bold numbers indicate a value <0.2 and thereby added into multivariable analysis.

‡ Bold numbers indicate statistical significance.

ERCP indicates endoscopic retrograde cholangiopancreatography; IPMN, intraductal papillary mucinous neoplasm; MCN, mucinous cystic neoplasm; OR, odds ratio; PTCD, percutaneous transhepatic cholangiography and drainage; SPN, solid pseudopapillary neoplasm.

**TABLE 3:** Predictors of IO in Patients With PDAC

	Univariable analysis [OR (95% CI)]	*P* ^†^	Multivariable analysis^*^ [OR (95% CI)]	*P* ^‡^
Age	0.99 (0.99–0.99)	**<0.001**	0.99 (0.99–0.99)	**0.002**
Female	1.22 (1.13–1.32)	**<0.001**	1.23 (1.14–1.33)	**<0.001**
ASA ≥3	0.79 (0.72–0.86)	**<0.001**	0.83 (0.75–0.91)	**<0.001**
Heart failure	0.91 (0.75–1.09)	0.313		
COPD	0.72 (0.60–0.85)	**<0.001**	0.74 (0.62–0.88)	**<0.001**
Diabetes mellitus	0.95 (0.87–1.03)	0.212		
Registry				
North America	Reference		Reference	
Germany	0.97 (0.88–1.07)	0.563	1.12 (1.01–1.26)	**0.032**
The Netherlands	1.15 (0.99–1.33)	**0.055**	1.19 (1.02–1.39)	**0.027**
Sweden	1.12 (0.91–1.40)	0.267	1.25 (0.99–1.57)	**0.055**
BMI	0.99 (0.99–0.99)	**0.034**	0.99 (0.99–0.99)	**0.036**
Performance status				
Independent	Reference		Reference	
Partially dependent	0.58 (0.44–0.77)	**<0.001**	0.61 (0.46–0.81)	**<0.001**
Fully dependent	0.05 (0.01–0.40)	**0.004**	0.05 (0.01–0.43)	**0.006**
Biliary drainage				
No	Reference		Reference	
Yes – ERCP	1.23 (1.14–1.33)	**<0.001**	1.22 (1.12–1.32)	**<0.001**
Yes – PTCD	0.96 (0.76–1.21)	0.733	0.98 (0.77–1.25)	0.867
Operation year	0.99 (0.95–1.04)	0.787		
Operative approach				
Open	Reference			
Minimally invasive	1.16 (0.97–1.49)	**0.102**		
Other	0.82 (0.59–1.13)	0.444		
Vascular resection				
No	Reference		Reference	
Vein	0.93 (0.85–1.02)	**0.123**	0.85 (0.77–0.93)	**<0.001**
Artery	0.82 (0.61–1.90)	**0.171**	0.81 (0.60–1.08)	0.154
Vein and artery	0.73 (0.58–0.92)	**0.007**	0.71 (0.57–0.90)	**0.004**
Neoadjuvant chemotherapy	1.46 (1.35–1.59)	<0.001	1.59 (1.36–1.62)	**<0.001**

* Multivariable analysis in 11,402 patients, no deletions due to missing values (on the imputed set).

† Bold numbers indicate a value <0.2 and thereby added into multivariable analysis.

‡ Bold numbers indicate statistical significance.

ERCP indicates endoscopic retrograde cholangiopancreatography; OR, odds ratio; PTCD, percutaneous transhepatic cholangiography and drainage.

## DISCUSSION

This transatlantic study proposed IO as a novel composite outcome measure in pancreatic surgery, defined by the absence of all 6 parameters: in-hospital mortality, severe complications –Clavien-Dindo ≥3, POPF – ISGPS classification grade B/C, reoperation, hospital stay >75th percentile, and readmission. IO was achieved in 54% of all patients, with an ALD of 3% among countries. This novel composite outcome measure, harmonizing the 2 previously reported composite outcome measures, is proposed to be used to audit and compare performance or quality of care between and within centers and countries.

The previous initial study reporting on optimal pancreatic surgery identified a rate of 57% in patients after PD in North America, whereas the initial study reporting on textbook outcome in The Netherlands identified a rate of 58% in patients after PD.^8,9^ Even though both these definitions are considered valid, the use of different definitions impedes the comparison of results, emphasizing the necessity of a consensus on the definition.^20^ Components of the original optimal pancreatic surgery and textbook outcome composite measures are included in the new IO definition, see Supplemental Digital Content 1, Table 2 (http://links.lww.com/SLA/E774).^8,9^ A recent systematic review evaluating textbook outcome in hepatopancreatobiliary surgery, advised that future definitions should include mortality, complications, LOS, and readmission.^21^ All these themes are included in the new definition of IO (Supplemental Digital Content 1, Table 2, http://links.lww.com/SLA/E774). Within the current study, 54% of patients achieved IO, which is comparable to the previous studies (57% optimal pancreatic surgery, 59% and 59% textbook outcome). Comparable results of IO with the previous definitions, and the adherence to the recommendations made within the systematic review, emphasize the utility of this new IO definition.

Composite outcome measures have been used to evaluate quality of surgical care, as individual event rates such as mortality are often too low to reliably measure hospital quality, and a single parameter often does not reflect the multidimensional aspect of the surgical outcome.^22,23^ This issue is illustrated by the results of the current study, in which individual components of IO differ considerably among countries (ALD: 2%–12%, RLD: 1.1–3.6), while the composite IO differs less (ALD: 3%, RLD: 1.1). One caution with composite outcomes is that outcome indicators can be interrelated.^24^ For example, a previous GAPASURG study showed that the country with the longest LOS after pancreatic surgery (16.0 days), had the lowest rate of readmissions (8.3%).^13^ Whereas the patients with the shortest LOS (9.0 days) had significantly higher readmission rates (16.5%). To minimize the influence of these effects, a prolonged LOS per country individually as a LOS >75 percentile.

The individual component which contributed the most (28%) to not achieving IO was ‘severe complications’, additionally showing the largest absolute differences (12%). Patients in Sweden had the fewest complications (20%), whereas the severe complication rates in Germany and The Netherlands were >10% higher. This finding could be explained by large efforts in centralization made in Sweden, with currently only 6 centers performing pancreatic surgery, as centralization is known to improve outcomes in pancreatic surgery.^4,25^ The relative largest differences are seen within in-hospital mortality (RLD: 3.6), with the lowest in-hospital mortality in North American patients. The good scores within in-hospital mortality may be explained by lower rates of failure to rescue (FTR; in-hospital mortality after major complications) in the North American patients, which has been described in a previous GAPASURG study comparing FTR in North American and European patients (5% vs 12%).^26^ The discrepancy of large differences among countries in FTR, and only minimal differences in IO, emphasizes that these outcomes complement each other. As mortality after pancreatic surgery is low, IO provides a way to evaluate outcomes above and beyond mortality and morbidity.

In this previous study, management of complications by radiologic drainage was associated with lower rates of FTR, and reoperation was associated with higher rates of FTR.^26^ The importance of radiological drainage to improve FTR was confirmed in the PORSCH trial, showing that an algorithm for early recognition and proactive management of postoperative complications (preferably by percutaneous drainage) after pancreatic surgery reduces 90-day mortality almost 50% (from 5% to 3%).^27^ Within the current study the reoperation rates in North America were lower (6%), compared with the European countries (Germany: 17%, The Netherlands 8%, Sweden 9%, ALD: 11.0%). Most likely this strategy does not only attribute to the lower rates of FTR, but also to the shorter LOS in North American patients. As it is well-known that patients undergoing reoperation have longer hospital stay compared with those who underwent radiologic drainage.^28^ Therefore, this study also emphasizes the need to focus on (early) radiologic drainage to improve outcomes, and eventually increase the rates of IO.

The current study identified age, sex, BMI, absence of COPD as a comorbidity, performance status, ASA score, preoperative biliary drainage, absence of vascular resection, and histologic diagnosis to be independently associated with IO. In the subgroup of patients with PDAC, neoadjuvant therapy, and country were additionally associated with IO. Patients with benign or premalignant neoplasms more often have a soft pancreas and nondilated pancreatic duct, which are both well-known risk factors for pancreatic fistula and intra-abdominal infections.^29^ Only a few studies describe the increased risk of ampullary tumors, especially duodenal carcinoma, on complications.^30,31^ In addition, neoadjuvant chemoradiotherapy therapy in patients with PDAC which is associated with better IO in the present study, reduces the risk of POPF and could thus improve the rate of IO.^32,33^ Among others, within a retrospective study of 13,257 patients, neoadjuvant chemotherapy was associated with reduced severe morbidity, POPF, organ-space infections, percutaneous drainage, reoperation, and prolonged LOS.^34^ However, this effect could also be due to selection bias, as the present study did not include patients who did not undergo pancreatic resection due to progression or clinical deterioration after neoadjuvant chemotherapy. The use of minimally invasive surgery was not associated with IO. This observation was comparable with a previous study evaluating the influence of minimally invasive PD on textbook oncological outcome between 2010 and 2015, which showed no difference among the 2 groups (23.5% vs 24.7%, *P*=0.28).^35^

One limitation of this analysis is, that not all variables are registered identically within all registries. Even though some variables are defined equally, their scoring still differs among countries. For example, ASA score is known to be associated with significant inter-rater variability.^36^ This is especially evident in the present study. To address this discrepancy, the MILESTONE-2 project has been initiated: a survey to gain knowledge on the extent of the variation in ASA classification within hepatopancreatobiliary surgery and identification of the underlying causes. Moreover, some variables which had to be recategorized to be able to compare results may account for some of the discrepancies. For example, due to the difference in use of TNM classification, Sweden has different numbers for stage 2B and 3 compared with the other countries, even though vascular resection rates are comparable. Therefore, vascular resections were used as a surrogate to define the extent of the tumor invasion in multivariable analysis, as this variable registered equally in all registries. To be able to accurately compare results, efforts are being taken to further harmonize the registries.

The chance of achieving IO was slightly higher in NSQIP, representing North America, compared with the European registries. Nevertheless, in multivariable analysis country is not independently associated with outcomes, and in the nonimputed set surgery being performed in Sweden is associated with improved outcomes. Unfortunately, in the GAPASURG dataset, no information on surgeon and center volume was available. This is relevant since in the NSQIP a larger proportion of high-volume centers may participate as compared with the nationwide (mandatory) audits, and surgeon experience and volume probably plays a role in achieving IO. A study including 20,902 patients after pancreatic resection derived from Medicare insurance files showed that patients after pancreatic resection by a top-quartile volume surgeon were more likely to achieve textbook outcome (70% vs 63%), whereas a resection by a low-quartile volume surgeon caused a 46% rate of textbook outcome.^37^ Multiple other studies investigating “oncological textbook outcome,” showed a clear association of higher surgical case volume and better outcomes.^38^

The results of this study should be interpreted in light of several limitations. First, the results of this study can be influenced by the difference in the structure of the registries, most importantly voluntary and multicenter (North America and Germany) versus mandatory and national (The Netherlands and Sweden), the frequency of auditing, and the duration of follow-up. Details on the differences among the registries are described in our previous study and within Supplemental Digital Content 1, Table 1 (http://links.lww.com/SLA/E774). Second, besides LOS, no information on discharge criteria is available in the different registries, including whether patients are discharged to their home or a special-care facility. This issue can bias the results, which is illustrated by the variation within LOS (between 8 and 16 days). Nevertheless, correction per country took place by taking the 75th percentile of the registry’s results as a cutoff. Third, this study only focused on the short-term (surgical) outcomes of patients who underwent PD, it would be worthwhile for future studies to assess the impact of IO on longer term (eg, 90 days) and oncological outcomes. Fourth, due to the large dataset, small differences become statistically significant, whereas they might not be clinically relevant. Therefore, ALD and RLD were also described in this study. Nevertheless, this study is a transatlantic evaluation of >20,000 patients who underwent PD, providing a novel definition of IO, including real-world outcomes.

In conclusion, the novel definition of IO in pancreatic surgery includes the absence of in-hospital mortality, severe complications – Clavien-Dindo ≥3, POPF – ISGPS grade B/C, reoperation, hospital stay >75th percentile, and readmission. Within this transatlantic analysis, IO was achieved in 54% of all patients, with an ALD of 3% among countries. This new definition can be used for auditing and comparing the quality of care after PD between and within centers and countries.

## Supplementary Material

SUPPLEMENTARY MATERIAL

## DISCUSSANT

### Frederik Berrevoet (Ghent, Belgium)

I would like to congratulate you on this transatlantic analysis of a combined composite outcome measure for quality evaluation of pancreaticoduodenectomy (PD). You performed a retrospective multinational registry evaluation after identification of a set of so-called ideal outcome (IO) measures in over 21,000 patients from different countries and continents. Both the number of patients and the different origin of the data strengthen the value of this analysis. The combination of various outcome parameters from the so-called “textbook outcome” and “optimal pancreatic surgery” is a very interesting and valuable merge that might, indeed, help to audit and compare outcome after PD worldwide. The study seems to be performed accurately and you could clearly show the various rates of IO in different countries.

First, in your analysis, and specifically in the discussion, you already highlighted some of the limitations of your work. I would like you to comment more extensively on the role of surgical quality versus overall oncological quality. How do you evaluate the role of the different healthcare systems represented in your study? In other words, could the results be biased by the fact that only in-hospital mortality is reported, and not 30- or 90-day mortality? In this regard, how do you evaluate length of stay as a valuable outcome measure in comparing various healthcare systems, as this is the individual component with the most variation (14%)?

Second, in the same regard, do you think that the “selection” of registration centers, in both the US and Germany, might have significantly biased your results? Do you have an insight into how much of the total number of PDs in the US and Germany are registered, and consequently, analyzed here? Do you believe this selection bias might undermine the usefulness of comparing data from other countries, as this cannot be “real world data”?

Third, how do you look at the separate analysis of the minimally invasive surgical procedures you performed with this registry data? As we have also observed in Belgium, the numbers of PD by MIS remain very low, and the results of your analysis are not associated with improved rates of achieving IO. Should we insist on promoting and pushing surgeons to perform minimally invasive PD?

Finally, another point of discussion could be how you see the role of evaluating post-discharge criteria for high-quality oncological care in PD, compared to the quite strict surgical care described here? It is very well-known that adjuvant therapy is influencing overall survival of patients with pancreatic adenocarcinoma, and therefore, this seems a valuable outcome measure. It is also indirectly related to 30d and 90d mortality. Can you please provide your opinion on this?

### Response From Simone Augustinus (Amsterdam, The Netherlands)

Thank you for your questions. I want to begin with the first and final questions combined, as they both concern the oncological outcomes and the more long-term results. Unfortunately, the registries only include surgical and short-term variables. We don’t have the long-term data available. However, we are trying to also include the oncological registries. Hopefully, in this way, we will be able to adequately compare the long-term results and add these to our transatlantic comparisons.

Regarding the different discharge criteria and length of stay, it is important to use the 75th percentile of the length of stay of the individual country. This will minimize the influence of the different discharge criteria, even though the readmission rate will still differ per hospital, potentially influencing the results. That’s why it’s important to have the composite outcome, as you include both the length of stay and readmission, providing a more global overview of performance.

Moreover, regarding minimally invasive surgery, your remark is very interesting. The numbers of minimal invasive surgery within our dataset are low. Between 2018 and 2020, the overall rate of minimally invasive surgery was only 7% of the total registry. Possibly, this indicates that it is not related. However, we are currently writing a paper to investigate the use of minimally invasive surgery in all the registries, as it tends to differ greatly. For example, in Sweden, they have just started to perform minimally invasive surgery, and in this set, there are only 20 Whipples performed in Sweden. On the other hand, if you look at The Netherlands, 19% of the patients are operated on using minimally invasive surgery. So, hopefully, in the next paper, we can elaborate on this.

Finally, regarding the international multicenter registries, we acknowledge that this is a limitation, and we have to take this into account in our analysis. In Germany, around 20% of all Whipples were included in our registries, which is relatively low; however, in North America, 65-70% of all Whipples were included. I think it’s still real-world data because all Whipples within the hospitals included in the study had to be registered.

### Keith D. Lillemoe (Boston, United States)

Congratulations on a very nice presentation. I would just like to acknowledge that the group from Amsterdam, and probably, The Netherlands as a whole, is the most collaborative group I’ve ever seen, so it’s not a surprise that they are leading this effort. However, as a surgical editor and pancreatic surgeon, I’m going to question the overall value of such analyses. At least once a month at *Annals of Surgery*, I receive a paper on “Textbook Outcomes” on literally every topic. In my opinion, surgical outcome still comes down to the surgeon, the patient, the operation, and more often, the decision-making. How are we to use this data, other than to say that we either met or failed to meet the ideal outcome? Will our patients consider us to be failures if we don’t do meet these standards? Will it get to the point where we’re all judged by the fact that we’re not reaching these outcomes? As you pointed out, big data has a lot of flaws and can be hard to translate to a specific patient. So, please tell me how to best use their data?

### Response From Simone Augustinus (Amsterdam, The Netherlands)

Thank you for your initial compliment and interesting question. I agree with what you say, but as you know from the number of papers you receive from multiple countries, we are faced with many different results and it is difficult to interpret them, due to the different structures of care. I think that if people also use the ideal outcome in their papers, then it will be much easier to compare the results among countries. You will still spot individual differences, but you will also obtain the broader picture. I don’t think it’s just about patient information and the measurement of care; it’s also about adequately comparing outcomes between countries. For example, if you compare a retrospective study from The Netherlands with one from North America, which both include the ideal outcome, then it’s easier to compare the results, as all parameters are taken into account.

### Michael Kerin (Galway, Ireland)

I enjoyed this paper very much. Do you have any insights into ideal surgeon and unit volume, in order to provide the ideal outcome?

### Response From Simone Augustinus (Amsterdam, The Netherlands)

We were also interested in this point. Unfortunately, the current dataset doesn’t include volume. However, we now have volume data for the Swedish, Dutch, and German registries. We didn’t have it at the time of writing this paper, but we will be including it in the next one. So, hopefully, we can provide more insight into this in our next paper to determine the benchmarks, for example. We do expect to see better outcomes with higher volumes.
