# Supplementary material for: Ideal Outcome After Pancreatoduodenectomy: A Transatlantic Evaluation of a Harmonized Composite Outcome Measure
Source: Ann Surg. 2023 Jul 21;278(5):740–7. doi: 10.1097/SLA.0000000000006037 (PMC10549886; doi:10.1097/SLA.0000000000006037)
Supplement: SUPPLEMENTARY MATERIAL [file sla-278-00740-s001.docx]

**Supplementary Table 1**. Most important differences in registry design

| **Theme** | **North America** | **Germany** | **The Netherlands** | **Sweden** |
| --- | --- | --- | --- | --- |
| **Design** | Multicenter | Multicenter | Nationwide | Nationwide |
| **Participation** | Voluntary | Voluntary | Mandatory | Mandatory |
| **Auditing** | No auditing, but data-entry by independent reviewers* | Yearly auditing | Auditing performed once (2017) | Bi-annual auditing |
| **Follow-up IO components** | 30-day | 30-day and in-hospital | 30-day and in-hospital | 30-day and in-hospital |

IO: ideal outcome. *The Surgical Clinical Reviewers undergo annual certification testing to assure inter-rater reliability. They are guided by published variable definitions, annual courses, quarterly webinars and daily variable question support.

**Supplementary Table 2**. Definition of Ideal Outcome derived from Optimal Pancreatic Surgery and Textbook Outcome

| **Theme** | **Optimal Pancreatic Surgery** | **Textbook Outcome** | **Ideal Outcome** |
| --- | --- | --- | --- |
| Mortality | Postoperative mortality | In-hospital mortality | In-hospital mortality |
| Complications | Serious morbidity | Severe complications (Clavien Dino ≥3) | Severe complications (Clavien Dino ≥3) |
|  | Percutaneous drainage |  |  |
| Pancreatic specific complications |  | Postoperative Pancreatic Fistula (Grade B/C*) | Postoperative Pancreatic Fistula (Grade B/C*) |
|  |  | Bile leak |  |
|  |  | Postpancreatectomy hemorrhage (Grade B/C*) |  |
| Reoperation | Reoperation |  | Reoperation |
| Length of stay | Length of stay^#^ (> 75 percentile) |  | Length of stay^#^ (> 75 percentile) |
| Readmission | Readmission | Readmission | Readmission |

*ISGPS: International Study Group of Pancreatic Surgery classification.

^#^Length of hospital stay >75^th^ percentile for the study cohort. This can be assessed in a single center cohort but also in a multicenter or nationwide cohort. For the present study the 75^th^ percentile was determined per audit, so for each of the 4 audits.

**Supplementary Table 3**. Predictors of Ideal Outcome: sensitivity analysis of non-imputed set

|  | **Univariable analysis  OR (95%CI)** | **P-value^a^** | **Multivariable analysis* OR (95% CI)** | **P-value^b^** |
| --- | --- | --- | --- | --- |
| Age | 0.99 (0.99-0.99) | **<0.001** | 0.99 (0.99-0.99) | **<0.001** |
| Female | 1.29 (1.22-1.37) | **<0.001** | 1.28 (1.21-1.36) | **<0.001** |
| ASA ≥ 3 | 0.89 (0.84-0.94) | **<0.001** | 0.89 (0.82-0.95) | **<0.001** |
| Heart failure | 0.90 (0.78-1.04) | **0.165** |  |  |
| COPD | 0.72 (0.63-0.81) | **<0.001** | 0.74 (0.65-0.85) | **<0.001** |
| Diabetes mellitus | 1.04 (0.97-1.11) | 0.235 |  |  |
| Registry  North America  Germany  the Netherlands  Sweden | Reference  0.92 (0.85-0.98) 0.88 (0.80-0.96) 0.98 (0.95-1.12) | **0.015 0.005 0.744** | Reference 0.94 (0.86-1.01) 0.97 (0.87-1.11) 1.19 (1.01-1.40) | 0.108 0.737 **0.033** |
| BMI | 0.99 (0.99-0.99) | **<0.001** | 0.99 (0.99-0.99) | **<0.001** |
| Performance status  Independent  Partially dependent  Fully dependent | Reference 0.60 (0.49-0.73) 0.14 (0.05-0.35) | **<0.001 <0.001** | Reference 0.64 (0.53-0.79) 0.17 (0.79-1.19) | **<0.001 <0.001** |
| Biliary drainage  No  Yes – ERCP  Yes – PTCD | Reference  1.36 (1.28-1.43) 1.03 (0.86-1.24) | **<0.001** 0.736 | Reference 1.25 (1.17-1.33) 0.97 (0.79-1.19) | **<0.001** 0.801 |
| Operation year | 0.99 (0.96-1.03) | 0.612 |  |  |
| Minimally invasive surgery  No  Yes   Other | Reference 1.01 (0.91-1.13) 0.82 (0.64-1.05) | 0.823 **0.117** |  |  |
| Vascular resection  No  Vein  Artery  Vein and artery | Reference 1.08 (0.99-1.17) 0.87 (0.69-1.08) 0.83 (0.69-1.01) | **0.072** 0.212 **0.058** | Reference 0.91 (0.84-0.99) 0.83 (0.66-1.05) 0.73 (0.59-0.89) | **0.047** 0.122 **0.002** |
| Histological diagnosis  Pancreatic adenocarcinoma  Distal cholangiocarcinoma  Ampullary carcinoma  Duodenal carcinoma  Neuroendocrine tumor  IPMN  MCN / serous cystadenoma  Chronic pancreatitis  SPN  Intestinal adenoma  Other | Reference  0.56 (0.49-0.64) 0.71 (0.65-0.79) 0.43 (0.37-0.50) 0.59 (0.52-0.67) 0.73 (0.65-0.82) 0.63 (0.49-0.79) 0.91 (0.79-1.05) 0.57 (0.36-0.89) 0.41 (0.27-0.63) 0.56 (0.51-0.63) | **<0.001 <0.001 <0.001 <0.001 <0.001 <0.001 0.198 0.016 <0.001 <0.001** | Reference 0.55 (0.48-0.63) 0.70 (0.63-0.78) 0.45 (0.38-0.54) 0.58 (0.51-0.66) 0.78 (0.68-0.88) 0.65 (0.51-0.84) 0.89 (0.77-1.04) 0.40 (0.24-0.66) 0.47 (0.29-0.78) 0.58 (0.52-0.64) | **<0.001 <0.001 <0.001 <0.001 <0.001 <0.001** 0.157 **<0.001 0.004 <0.001** |

*Multivariable analysis in 18,745 patients, 2,291 deleted due to missing values. ^a^Bold numbers indicate a value <0.2 and thereby added into multivariable analysis. ^b^Bold numbers indicate statistical significance.
