# Supplementary material for: Ideal Outcome After Pancreatoduodenectomy: A Transatlantic Evaluation of a Harmonized Composite Outcome Measure
Source: Ann Surg. 2023 Jul 21;278(5):740–7. doi: 10.1097/SLA.0000000000006037 (PMC10549886; doi:10.1097/SLA.0000000000006037)
Supplement: SUPPLEMENTARY MATERIAL [file sla-278-00740-s002.docx]

**Table 2.** Baseline characteristics

|  | **North America (n = 13,883)** | **Germany (n = 3,964)** | **The Netherlands (n = 2,188)** | **Sweden (n = 1,001)** | **Total (n =21,036)** | **ALD** | **RLD** |
| --- | --- | --- | --- | --- | --- | --- | --- |
| Age  *Missing* | 67.0 (59.0-73.0) *0* | 69.0 (60.0-77.0) *0* | 69.0 (61.0-75.0) *2* | 70.0 (64.0-75.0) *185* | 68.0 (60.0-74.0) *187* | 3.0 | 1.03 |
| Female  *Missing* | 6,499 (47%) *0* | 1,686 (43%) *0* | 995 (46%) *3* | 475 (47%) *0* | 9,655 (46%) *3* | 4% | 1.09 |
| ASA ≥3  *Missing* | 11,284 (81%) *6* | 2,130 (54%) *0* | 729 (34%) *34* | 286 (29%) *7* | 14,429 (69%) *47* | 52% | 2.79 |
| Heart failure  *Missing* | 57 (<1%) *0* | 528 (14%) *0* | 72 (4%) *154* | 83 (8%) *6* | 832 (4%) *238* | 13% | 14.0 |
| COPD  *Missing* | 525 (4%) *0* | 190 (5%) *0* | 284 (14%) *154* | 220 (22%) *6* | 1,082 (5%) *160* | 18% | 5.50 |
| Diabetes mellitus  *Missing* | 3,793 (27%) *0* | 998 (25%) *1* | 481 (24%) *154* | 220 (22%) *6* | 5,482 (26%) *161* | 5% | 1.23 |
| BMI  *Missing* | 26.5 (23.3-30.4) *83* | 24.8 (22.3-27.8) *13* | 24.8 (22.3-27.7) *39* | 25.0 (22.5-27.9) *28* | 25.8 (23.0-19.5) *163* | 1.7 | 1.07 |
| Performance status  Independent  Partially dependent  Fully dependent  *Missing* | 13,749 (99%) 115 (1%) 9 (<1%) *10* | 3,785 (96%) 156 (4%) 22 (<1%) *1* | 1,599 (93%) 114 (7%) 1 (<1%) *474* | 923 (93%) 69 (7%) 1 (<1%) *8* | 20,056 (98%) 454 (2%) 473 (<1%) *493* | 6% 6% 0% | 1.06 7.00 0.00 |
| Preoperative Biliary drainage  No  Yes – ERCP  Yes – PTCD   *Missing* | 5,874 (44%) 6,949 (53%) 377 (3%) *683* | 2,428 (61%) 1,535 (39%) *Not registered* *1* | 932 (44%) 1,108 (52%) 96 (5%) *52* | 463 (47%) 532 (53%) *Not registered* *6* | 9,697 (48%) 10,124 (50%) 473 (2%) *742* | 17% 14% 2% | 1.39 1.36 NA |
| Neoadjuvant chemotherapy*  *Missing* | 3,261 (40%) *35* | 143 (7%) *1* | 259 (30%) *20* | 44 (11%) *1* | 3,707 (33%) *57* | 33% | 5.71 |

ALD: Absolute largest difference. RLD: relative largest difference. ASA: American Society of Anesthesiologist Physical Status Score. BMI: body mass index. ERCP: Endoscopic retrograde cholangiopancreatography. PTCD: percutaneous transhepatic biliary drainage. NA: not applicable, because not available in all countries. Numbers are depicted as valid percentages. *In patients with pancreatic adenocarcinoma (n=11,402), including chemo radiation in North America, Germany, the Netherlands.
