# Supplementary material for: Ideal Outcome After Pancreatoduodenectomy: A Transatlantic Evaluation of a Harmonized Composite Outcome Measure
Source: Ann Surg. 2023 Jul 21;278(5):740–7. doi: 10.1097/SLA.0000000000006037 (PMC10549886; doi:10.1097/SLA.0000000000006037)
Supplement: SUPPLEMENTARY MATERIAL [file sla-278-00740-s003.docx]

**Table 3.** Per-operative and histological characteristics

|  | **North America (n = 13,883)** | **Germany (n = 3,964)** | **The Netherlands (n = 2,188)** | **Sweden (n = 1,001)** | **Total (n =21,036)** | **ALD** | **RLD** |
| --- | --- | --- | --- | --- | --- | --- | --- |
| Surgical approach  Open (incl. conversion)  Minimally invasive surgery  Other (incl. hybrid)  *Missing* | 12,753 (92%) 860 (6%) 266 (2%) *191* | 3,870 (98%) 89 (2%) 0 *5* | 1,734 (81%) 411 (19%) 0 *43* | 613 (100%) 1 (<1%) 0 *387* | 18,970 (92%) 1,361 (7%) 266 (1%) *439* | 19% 18% 2% | 1.23 19.00 NA |
| Type of PD  Pylorus preserving PD  Pylorus resecting PD / classic   Whipple | 4700 (34%) 9183 (66%) | 2832 (71%) 1,132 (29%) | 917 (42%) 1271 (58%) | 253 (25%) 748 (75%) | 8,702 (41%) 12,334 (59%) | 46% 46% | 2.84 2.59 |
| Vascular resection  No  Vein  Artery  Vein and artery  *Missing* | 11,068 (81%) 1,890 (14%) 290 (2%) 444 (3%) *191* | 3,455 (87%) 493 (12%) 10 (<1%) 6 (<1%) *0* | 1,825 (84%) 312 (14%) 25 (1%) 6 (<1%) *20* | 814 (81%) 176 (18%) 6 (<1%) 3 (<1%) *2* | 17,162 (82%) 2,871 (14%) 331 (2%) 459 (2%) *213* | 6% 6% 1% 2% | 1.07 1.50 2.00 3.00 |
| Length of stay, median (IQR) *Missing* | 8.0 (6.0-12.0) *478* | 16.0 (12.0-24.0) *22* | 11.0 (8.0-19.0) *14* | 13.0 (9.0-19.0) *108* | 9.0 (7.0-12.0) *622* | 8.0 | 1.23 |
| Histological diagnosis  Pancreatic adenocarcinoma  Ampullary carcinoma  Distal cholangiocarcinoma  Duodenal carcinoma  Neuroendocrine tumor  IPMN  MCN / serous cystadenoma  Chronic pancreatitis  SPN  Intestinal adenoma  Other  *Missing* | 8,097 (59%) 1,058 (8%) 432 (3%) 433 (3%) 837 (6%) 854 (6%) 217 (2%) 421 (3%) 47 (<1%) 0 1,250 (9%) *237* | 2,010 (51%) 343 (9%) 316 (8%) 104 (3%) 120 (3%) 235 (6%) 59 (2%) 380 (10%) 19 (1%) 0 33 (9%) *46* | 884 (41%) 312 (14%) 273 (13%) 147 (7%) 97 (5%) 168 (8%) 14 (1%) 59 (3%) 10 (1%) 62 (3%) 143 (7%) *19* | 411 (47%) 131 (15%) 120 (14%) 58 (7%) 19 (2%) 20 (2%) 0  26 (3%) 0  31 (4%) 65 (7%) *120* | 11,402 (55%) 1,844 (9%) 1,141 (5%) 742 (4%) 1,073 (5%) 1,277 (6%) 290 (1%) 886 (4%) 75 (<1%) 93 (1%) 1,791 (9%) *422* | 18% 7% 11% 4% 4% 6% 2% 7% 1% 4% 2% | 1.44 1.88 4.67 2.33 3.00 4.00 NA  3.33 NA NA 1.29 |
| Tumor stage*   Stage 0   Stage 1A  Stage 1B  Stage 2A  Stage 2B  Stage 3  Stage 4  *Missing* | 108 (1%) 1,060 (14%) 1,202 (15%) 392 (5%) 3,341 (43%) 1,602 (20%) 135 (2%) *257* | 19 (1%) 133 (7%) 325 (16%) 104 (5%) 719 (36%) 629 (31%) 77 (4%) *4* | 0  88 (10%) 143 (17%) 26 (3%) 324 (37%) 263 (30%) 21 (2%) *19* | 6 (2%) 13 (3%) 25 (7%) 27 (7%) 276 (73%) 9 (2%) 24 (6%) *31* | 133 (1%) 1,294 (12%) 1,695 (15%) 549 (5%) 4,660 (42%) 2,503 (23%) 257 (2%) *311* | 2% 11% 9% 4% 37% 29% 4% | NA 4.67 2.29 2.33 2.03 15.50  3.00 |

ALD: Absolute largest difference. RLD: relative largest difference. PD: pancreatoduodenectomy. IPMN: Intraductal papillary mucinous neoplasm. MCN: mucinous cystic neoplasms. SPN: Solid pseudopapillary neoplasm. Numbers are depicted as valid percentages. When no missing are available, missing are not described. *In patients with pancreatic adenocarcinoma (n=11,402)
